# Strengthening the service experiences of women impacted by gambling-related intimate partner violence

**DOI:** 10.1186/s12889-022-13214-9

**Published:** 2022-04-14

**Authors:** Cathy O’Mullan, Nerilee Hing, Elaine Nuske, Helen Breen, Lydia Mainey

**Affiliations:** 1 Central Queensland University, University Drive, Bundaberg, QLD 4670 Australia; 2 Southern Cross University, Military Rd, East Lismore, NSW Australia; 3 Central Queensland University, Shield Street, Cairns, QLD 4870 Australia

**Keywords:** Gambling, Intimate partner Violence, Service provision, Help-seeking, Women

## Abstract

**Background:**

While problem gambling does not directly cause intimate partner violence (IPV), it exacerbates that violence significantly. Women experiencing both gambling harm and IPV often find themselves in challenging situations; furthermore, stigma and shame frequently act as barriers to seeking help from health and social service agencies. Despite the links between problem gambling and IPV, little is known about women’s experiences of using support services for both IPV and gambling related issues. This paper explores positive experiences of help-seeking for gambling-related IPV in Australia by adopting a strengths-based research approach.

**Methods:**

Qualitative, unstructured interviews were conducted for a larger study exploring the nature of the relationship between problem gambling and IPV. To gain new insights into the service experiences of women impacted by gambling related IPV, interviews with 48 women with lived experience of IPV relating to a male partner’s gambling, and 24 women with lived experience of IPV relating to their own gambling were reanalysed using thematic analysis.

**Results:**

Three themes emerged from the data signifying or demonstrating strength-based responses: ‘Commitment to Integrated and Collaborative Responses’; ‘Therapeutic Support’; and ‘Instrumental Support’. The themes highlight the importance of recognising the intersectionality of gambling related IPV and supporting the person ‘at the centre of the service’. Tangible and instrumental supports, such as emergency accommodation and financial assistance, were also central to the recovery process.

**Conclusion:**

Effective service responses are dependent on understanding how problem gambling and IPV intersect. Importantly, service providers must recognise and address the many facets of each woman’s situation and the shame associated with resolving interdependent and complex issues. Responding to the needs of women impacted by gambling related IPV requires both individual-level awareness and organisational support; recommendations to strengthen service provision are provided.

## Background

Intimate partner violence (IPV) against women is a pervasive public health problem, affecting approximately one in three women globally [1]. IPV has been defined as behaviour by an intimate partner or ex-partner that causes physical, psychological, emotional or sexual harm [1]. Economic abuse, defined as a pattern of control, exploitation or sabotage of money, finances and economic resources, has been a relatively ‘invisible’ form of IPV, but is now increasingly recognised [2]. Problem gambling is also a public health issue and is clearly and strongly linked with both the perpetration and victimisation of IPV, including economic abuse [2–4]. While prevalence figures vary, international studies report that a little over one-third of individuals with a gambling problem have been a victim of physical IPV or have perpetrated physical IPV in the previous 12 months [3]. In Australia, research undertaken by Dowling et al. [5], revealed 27% of gamblers attending a gambling help service had experienced physical violence. Existing research also recognises the relationship between gambling and IPV as bi-directional; problem gambling contributes to IPV and IPV contributes to problem gambling [6, 7]. As noted by Freytag et al. [8], IPV and problem gambling are significant public health problems in their own right, but they regularly co-occur and create an even more dangerous combination.

Problem gambling and IPV are both significantly gendered: problem gambling is twice as common among men compared to women [9], with gender also playing a clear role in IPV with a preponderance of male perpetrators and female victims [10, 11]. Of note, men are more likely to perpetrate IPV if they hold attitudes supporting gender inequality [10]. While experiences of gambling harm do not solely cause IPV, when the gendered drivers of violence are present, gambling problems and the associated stressors can intensify and exacerbate IPV [12]. IPV can co-occur with the perpetrator’s gambling problem and subsequent anger about gambling losses; or with the perpetrator’s anger about the victim’s gambling and associated stressors [7, 13]. Concerningly, some women impacted by IPV seek refuge in gambling venues as they often provide the only safe space to escape from a violent partner [12]. However, by using gambling venues as safe spaces, many women become trapped in a vicious cycle by chasing gambling losses; this cycle increases their vulnerability to IPV [14]. International studies and those undertaken in Australia, report higher IPV victimisation rates amongst women with a gambling problem than men [4, 5, 15]. Not surprisingly, women experiencing both gambling harm and IPV often find themselves in very complex and challenging situations.

Health and social support services can play a crucial role in preventing and managing gambling related IPV and its harmful health impacts. In Australia, however, delivering and managing these services is a difficult task; consequently, individuals with multiple needs face additional barriers exacerbated by navigating a complex service delivery system [16]. As highlighted by the Productivity Commission [17], every level of government is involved in funding or delivering human services, with access and availability of services differing between States and Territories. The existing system is based on public payments to primarily non-government service providers (nonprofit and profit), referred to as a ‘mixed economy of care’ [18]. While the level of funding assistance to service providers varies across Australia, users rarely face the full cost of service provision. However, who receives the funding, when and on what basis, is a significant driver of health and social support services outcomes [17].

It is widely recognised that front-line healthcare and social support workers can be IPV survivors’ first and only contact with professionals [19, 20], representing an opportunity for routine enquiry regarding other health-related issues. There is a consensus that health service providers should ask women about IPV, stay alert to possible signs and symptoms, provide health and social support, provide information on available resources, and co-ordinate timely referrals to other agencies [20]. These actions should ensure privacy and confidentiality in a supportive environment where women’s experiences are validated and their decisions respected [19]. Freytag et al. [8] noted that seeking safety, dignity, and respect for gambling-related IPV can be met with escalated violence and abuse; help-seeking efforts must be carefully and strategically planned to minimise unintended consequences. Crucially, women may choose to use non-DFV services such as relationship or family dispute resolution (FDR) services for IPV, or gambling help services where gambling is an issue [21]. In short, non-DFV service providers, including gambling help and financial support services, must be skilled at responding constructively to gambling related IPV as they are highly likely to see clients impacted by this issue.

The service experiences of women seeking help for IPV, particularly IPV with co-occurring health issues, are not always satisfactory [22, 23]. While stigma and shame have been widely reported as barriers to help-seeking [24, 25], organisational and individual level barriers within services have also been identified. Barriers facing service providers can include time constraints, victim-blaming attitudes, lack of professional development, limited referral options, and lack of specialist support [19, 22, 24]. Health and social service workers (outside of specialist and integrated DFV services) rarely ask about IPV, and service users are frequently reluctant to disclose in the absence of direct questioning [26, 27]. While studies have shown a high occurrence of IPV in family members seeking help for problem gambling issues [7], screening for problem gambling in community services and healthcare settings is ad-hoc, rarely happens or is at the discretion of individual practitioners [28, 29].Lack of integrated services and inadequate triage and referral systems have also been identified as significant barriers to effective service responses [27, 30]. This situation is highly problematic, given that non-DFV services may see a more significant volume of women impacted by IPV than specialist DFV services do [21]. Mason and colleagues [31] highlighted that specialist services and those with limited networks are not usually funded to screen for other issues beyond their specialisation and typically apply a narrow lens. Siloed responses are particularly problematic for women who have complex, interconnected needs.

Reducing gambling related IPV against women requires a multi-pronged approach that reduces problem gambling and gender inequality and simultaneously improves service responses to these issues. Applying a comprehensive public health approach to promote upstream factors that mitigate the burden of harm at a population level is critical [32]; the importance of addressing the determinants of IPV and problem gambling have been documented elsewhere [6, 10]. At an individual level, supporting women impacted by gambling-related violence is important, however, women’s experiences of seeking help remain unexplored. Although women’s experiences of help-seeking for IPV [19, 33] and for problem gambling [34, 35] have been widely documented in systematic reviews, women experiencing gambling related IPV face unique and challenging circumstances. Studies undertaken into family violence with gambling help-seeking populations [3, 36] highlight the need for better treatment and support services for women and call for further research to be undertaken on this issue. Capturing the lived experiences of women accessing services for gambling related IPV is important, as findings can be used to inform targeted public health interventions and tailored treatment.

This paper aims to explore the service experiences of women impacted by gambling related IPV in Australia; the paper explores positive experiences of help-seeking by adopting a strengths-based research approach. Strengths-based approaches concentrate on the inherent strengths of individuals, groups and organisations, and embrace asset-based approaches which are solution-focused rather than problem-focused [37]. To our knowledge, this is the first study of services experiences of women affected by gambling related IPV to take a strengths-based approach.

## Methods

Ethical approval for this study was obtained from Central Queensland Human Research Ethics Committee (Protocol code # 20,852).

### Design

In the present study, we draw upon qualitative data collected as part of a larger study funded by ANROWS (project number#) which explored the nature of the relationship between gambling and IPV against women by a male partner using a social-ecological approach. This approach explores complex interactions between various individual and contextual factors, paying explicit attention to these relations’ social, institutional, and cultural contexts [38]. Specific research questions included: “How does gambling by a male partner interact with his violence against his female partner?”; “How does gambling by a female partner interact with violence from her male partner?” The study focused on gambling related IPV by men against a female partner as this is the most common form of IPV linked to gambling [5, 39]. Adaptive grounded theory [40] and situational analysis [41] were used as the methodological approach; this combined approach facilitated a richer and more credible understanding of women’s experiences of gambling related IPV. The specific design and methods have been detailed elsewhere [12]. Using interview data from the larger study, we performed a secondary analysis of data to answer the following research question “How can health and social service agencies be strengthened to better address the needs of women seeking support for gambling-related IPV?” This analytical stage of the research specifically focused on reanalysing interviews from the preceding stages of the study: 48 transcripts from women with lived experience of IPV related to a male partner’s gambling; and 24 transcripts from women with lived experience of IPV related to their own gambling.

### Recruitment and sample

Women throughout Australia who had experienced IPV (including economic abuse), linked to their own or male partner’s gambling, were purposively recruited to the study. Recruitment and data collection were conducted simultaneously between July 2018 and June 2019. Recruitment occurred through professional networks and direct contact with support services (e.g., gambling help services, domestic violence services, financial counselling services, legal services, women’s health services, culturally specific services). To minimise risk, participants were offered multiple options for contacting the research team: online registration, telephone, email and SMS; participants were encouraged to use a pseudonym. A female project officer spoke with each woman to ensure they met the inclusion criteria and to arrange an interview.

The inclusion criteria for participants were: being aged 18 years or over, currently living in Australia, willing to consent to and participate in an interview, and having lived experience of IPV (including economic abuse), linked to their own or male partner’s gambling. In line with ethics requirements and approval, we recruited only women who had experienced gambling related IPV and received professional help for one or both of these issues.

### Data collection

After confirming informed consent, telephone interviews lasting 50–90 min were undertaken with each woman. Each interview was facilitated by a female researcher with experience in conducting qualitative research on sensitive topics. Upon reflection, the interviewers felt that sharing the same gender as participants helped to build rapport and facilitate open communication. Participants were asked to tell their story of how gambling and IPV had impacted on their life. Unstructured interviews were used to allow the women to focus on their lived experience, however, each interviewer used a set of potential prompts to clarify issues such as the type(s) of problematic gambling, the trajectory of the gambling and IPV, and people, groups, and organisations that may have helped or hindered the situation. Most participants provided detailed accounts of their experiences with minimal prompting, including their experiences of help-seeking. All interviews were audio-recorded, professionally transcribed, and deidentified before analysis.

### Data analysis

To answer the research question “How can health and social service agencies be strengthened to better address the needs of women seeking support for gambling related IPV?”, we performed a secondary analysis of the interview data following the six phases of thematic analysis suggested by Braun and Clarke [42]. The first phase involved the first author reading and rereading the transcripts and noting help-seeking experiences and service interactions. Following this phase, codes (words or short phrases to capture key ideas) were generated from the data set and noted by the first author. Codes were clustered together to create potential themes. Provisional themes were discussed with the second author and agreed upon at this stage. Once provisional themes were selected, the first (COM) and second authors (NH) reviewed, defined, and named the final themes and sub-themes. The final phase of analysis involved selecting appropriate data excerpts to use for the final manuscript.

## Findings

### Participant characteristics

In total, there were 48 women participants aged between 20 and 69 years experiencing gambling related IPV linked to a male partner’s gambling (pseudonym WMG). The majority (39/48) of these women resided in metropolitan areas across Australia, while nine lived in regional areas. There were 24 women participants aged between 20 and 69 years experiencing gambling related IPV linked to their own gambling (pseudonym WWG). Most women (19/24) resided in metropolitan areas (Table 1).

**Table 1:** Key characteristics of participants interviewed about gambling related IPV

	Age 20–29	Age 30–39	Age 40–49	Age 50–59	Age 60–69	Reside in metropolitan area	Reside in regional area
WWG *n* = 24	3	8	4	4	5	19	5
WMG *n* = 48	7	17	11	8	5	39	9

All participants reported experiencing multiple forms of IPV from a male partner; most often, the abuse was verbal, emotional and psychological, along with physical and, less commonly, sexual violence. The violence was relentless, typically escalated over time, and included psychological abuse, stalking and physical assault. Many women were subjected to near-continuous coercion and control and lived in continual fear of further violence. Of note, nearly all participants whose partner had a gambling problem described being subjected to severe economic abuse, including economic control, such as withholding money, and economic exploitation, such as stealing funds. Most women reported that their partner was abusive, misogynistic and controlling before the commencement or escalation of his (or her) gambling problem, reflecting gendered attitudes that underpin men’s violence against women [10].

IPV linked to the women’s gambling was bi-directional. Seven women (7/24) identified as having a gambling problem prior to their victimisation. They believed their experiences of IPV were exacerbated by the tension and conflict arising from their gambling. For most of these women, however, the IPV had preceded the development of their gambling problem. The temporal sequence of gambling and IPV was uncertain for several women, as both issues co-occurred and developed over time. Irrespective of whether the gambling problem initially preceded the IPV or vice versa, all the women were caught in a cycle of gambling and violence driven and reinforced by similar factors.

Our recruitment criteria meant that all women had accessed formal support for gambling or IPV. While it was difficult to discern the exact order or frequency of service usage due to memory recall issues and the unstructured nature of the interviews, women most sought assistance from DFV services, gambling, financial or relationship counsellors, GPs, and mental health professionals. Some also accessed hospitals, alcohol and other drug services, generic counselling services, and court-appointed social workers. In addition, some women subjected to severe economic abuse had substantial interactions with banks and other financial institutions to take back control of family finances or prevent further financial loss. Other agencies of significance included Centrelink (Australian Government agency providing social, health and child support payments and services), and crisis accommodation services. In summary, most women accessed multiple services depending on their issues and the capacity of services to meet their needs.

### Themes

Three major themes and six sub-themes were derived from the thematic analysis (Table 2). Each theme will be explored individually, using verbatim quotations from women with lived experience of IPV linked to a male partner’s gambling (WMG) and women with lived experience of IPV linked to their own gambling (WWG) to illustrate the concepts.

**Table 2:** List of themes and subthemes

	**Theme**	**Sub themes**
Theme 1	Commitment to Integrated and Collaborative Responses	• Individual level responses • Organisation level responses
Theme 2	Therapeutic Support	• Person-centred, trauma informed support •Group Support
Theme 3	Instrumental Support	• Support from DFV sector • Support from Gambling Help and Financial Counselling sector

### Theme 1 commitment to integrated and collaborative responses

#### Individual level responses

At the individual practitioner level, most women highlighted the importance of working with practitioners who understood and addressed the intersecting barriers faced by women seeking support for gambling related IPV. Women seeking help for issues relating to their own or their partner’s gambling may not seek help for IPV, and vice versa, hence practitioners need to be alert to both issues. As highlighted by one woman seeking help for problem gambling, women valued practitioners who were alert to indicators and able to offer skilled assistance or a warm referral:

*“She was helping – like she was being a financial adviser. And when she realised how much I have to pay for…it was a bit, you know, unfair, so she suggested to see a lady over there [domestic violence counsellor].”* (Narelle, WWG, age 20-29)

In many cases, women did not initially identify economic abuse as IPV, or recognise the links between problem gambling and IPV. Instead, individual practitioners who were attentive to these issues and sought to address both problems helped women gain new insights into their situation and receive appropriate support. As noted by one woman, who sought free financial counselling (as part of the Federal government’s problem gambling program), “…*it was the counsellor that first suggested that it was domestic violence…up until that point, I just couldn’t accept it, or couldn’t really believe it.”* (Stacey, WMG, age 30–39). Similarly, another woman found out about economic abuse through her financial counsellor, who suggested she seek support for IPV. Even though her partner stole from her and demanded her pay packet in front of customers, she noted: *“…I wasn’t even aware that finances were part of the abuse.” (*Janet, WMG, age 40–49).

Practitioners who were sensitive to the intersection of gambling and IPV were able to assist women experiencing both issues. For example, one woman with lived experience of IPV relating to her own gambling highlighted how a visit to a relationship counsellor at Relationships Australia (a community based, not for profit organisation) with her partner helped her understand that IPV was not acceptable in any situation and that IPV was an underlying cause of her gambling problem. When her partner used her gambling problem to justify his abusive behaviour, “… *the counsellor said to him ‘There is never any excuse to hit a woman, there are no buts’.”* (Amber, WWG, age 30–39). Assessment of the clients’ situation, and the identification of gambling as a trigger and reinforcer of IPV, ensured she was able to obtain appropriate support for both issues.

Adopting an integrated and holistic response ensures all major issues impacting detrimentally on the client are known from the start. While programs often work on single issues, for example, problem gambling or IPV victimisation, counsellors who were alert to multiple health issues were reported as being extremely helpful. This is illustrated in the following quote, where a social worker who was committed to addressing a woman’s gambling problem and IPV concurrently used a more holistic approach:

*“They said, “if there’s any issues you have, we would like you to tell us about them, so that we can help you with your life”. I told them everything. They said, “the more we know, the more we can assess whether we can help you.”* (Jacky, WWG, age 60-69)

#### Organisation level responses

At an organisational level, integrated services (e.g., those that offered IPV, gambling support and mental health services in the one agency), and services with strong external links to other services were valued by women. In many cases, this ensured that women did not have to continually repeat and thus re-live their traumatic story to every agency. For one woman, having Child Protection Services (a State-based government service), and Parenting Alive programs (private advocacy, education and counselling service) linked to her counselling was very helpful in supporting her and her children. Another woman also found that referrals between Child Protection Services, Act for Kids programs (an Australian charity providing free therapy services) and Journeys for Women (a free eight-week program for women impacted by violence), assisted in rebuilding her life with her young children. Several different services connected to her counselling helped one woman draw up a safety plan and eventually leave an abusive relationship:

*“ [We] went through everything and he [the counsellor] sort of prepared me and we talked about all the things that I’d need to consider…how to deal with it all, so that was a really crucial factor…I couldn’t have done any of it…if it weren’t for the counsellor…I thought I was working to try and change my partner and it ultimately got to the point where I felt uncomfortable enough that I wanted to change…I didn’t want to be in that situation anymore.”* (Jody, WMG, age 20-29)

As noted earlier, many women came to services with risks they were not aware of or did not have a name for. At an organisational level, structured risk screening tools seemed to offer an effective, non-threatening way for practitioners to ask about risk so that all major issues were identified. Several women spoke highly of service experiences which included a comprehensive assessment of health issues. Carol recounted a positive experience accessing a not for profit women’s health service for IPV related issues:

*“The first session was spent completing a screening tool which seemed to cover everything, you know, mental health, drugs, relationships as well as the DV. I was quite surprised they asked about gambling, I thought to myself “this service knows what they are doing”, and they didn’t judge or criticise me when I told them about my gambling.”* (Carol, WWG, age 40-49)

Finding free services, such as counselling and gambling help services, was particularly valued by women in this study. Economic abuse had left many women with no spare funds hence the cost and accessibility of some services often deterred women from seeking help. One woman explained how she was able to access free support through a gambling help service to help her cope and recover from gambling related IPV:

*“If I go through Relationships Australia about the gambling, I can get it for free, the counselling, whereas I couldn’t get relationship counselling for free. So, one of the reasons I went through the gambling avenue was because it was free.”* (Janey, WMG, age 30-39)

Protective service models that had a permanent base, such as Relationships Australia (a long standing, not for profit organisation) and Centrelink were found to be very helpful as they were perceived to have reliable and stable funding cycles. This meant that the women could anticipate some continuity of support in their recovery. By returning to the same service and not having to relive their abuse by repeating their story “…*because it takes about five sessions out of the ten sessions to get your family tree down, or whatnot, you know.”* (Skye, WMG, age 20–29); they could move forward with their healing.

### Theme 2 therapeutic support

#### Person-centred, trauma-informed support

Women who had very positive service experiences frequently spoke about the value of services being non-judgmental, sensitive to their situation, and trauma-informed. Women frequently talked about feeling guilty or responsible for their situation, irrespective of who had the gambling problem. They particularly valued being able to talk about their harms without being judged. As one woman who sought financial help for economic abuse noted:

*“The people I talk to and been in contact with, they were all wonderful. There was not a speck of judgment or criticism or anything that – they were all so understanding and really – you could just feel that they tried everything to help…”* (Noel, WMG, age 50-59)

Women also described being provided with trauma-informed support in favourable ways. Following several distressing sessions with a private psychologist, one young woman commenced treatment with a Head Space counsellor who tailored the sessions to her unique needs and circumstances (Head Space provide free mental health counselling to young people):

*“I met him the first time, and he said, “Is this useful?” Nobody ever asked me that before. They just said, “See you next week.” But he said, “Is this useful?” And I had to think about it, and I said, “Actually, yes. Yes, I like listening to you. I like talking to you”. And the other thing I felt about seeing him was I felt upbeat when I came out. I’d feel hopeful, and he would have given me things, interesting things, to try, that I could report on the next time I saw him.”* (Andrea, WWG, age 20-29)

In a similar vein, the following woman highlighted the value of person-centred approaches that focus on achieving personal aspirations and are tailored to her needs. Other women were also key advocates of moving beyond a crisis response, and supported the idea of long-term approaches to help recover from trauma:

*“.... there are some really good programs, I’m participating in one at the moment…where you actually participate in bush therapy and equine therapy, and they’re fantastic therapeutic processes. We need programs that are future focused…moving forward with and putting the trauma behind you.”* (Joe, WMG, age 50-59)

A person-centred approach is critical in the context of gambling related IPV. All women who identified as having a gambling problem spoke at length about the attraction of gambling venues and how gambling functions as an escape and a survival tool,”*…when the violence and the emotional abuse would erupt, I would leave the house, because I had no friends or family around me. So I would actually go the pokies and that’s where I would stay. I was never coming home. I didn’t want to be at home.”* (Sheila, WWG, age 60–69). Practitioners who were able to explore the role gambling played in helping to cope with or escape IPV, and who encouraged alternative solutions, were rated highly by women.

#### Group support

Sharing lived experiences of gambling related IPV through group counselling or a support group, was a crucial part of the healing process for many women. While these women were initially reluctant to attend group sessions (citing concerns about confidentiality or fear of discrimination), connecting with others in similar situations and sharing advice helped reduce their social and emotional isolation. One woman seeking help for IPV related to her partner’s gambling recalled:

*“I started sharing my story, and once I’d said enough of my story and I got all that out into the open…I was doing a lot of listening and I learnt from other people’s experiences.”* (Jacky, WWG, age 60-69)

As evidenced by the women’s stories, current conceptualisations of problem gambling are damaging and can impact help-seeking. For some women experiencing a gambling problem, attending a support group helped to lift the veil of secrecy, to challenge some of problem gambler stereotypes, and to reduce self-stigma:

*“That was a very therapeutic thing for me too because I was speaking with people and we were starting to realise that we were normal people. We weren’t people with two heads, this social, retrograde, homeless, toothless wonder who frankly what can you do to help, they’re buggered anyway.”* (Mandy, WWG, age 20-29)

Most women in the study struggled with the shame and stigma associated with gambling related IPV and frequently held themselves responsible for the situation. Several women benefited from attending Gam-Anon, a twelve-step program that holds peer support groups for family members affected by problem gambling. These women spoke highly of this experience and found that engaging with others in similar situations was extremely helpful:

*“Gam-Anon lets you get it out. That’s the wonderful thing in that, and these people…When I told my story to people who understood, oh god, it’s better than gold.”* (Anna, WMG, age 40-49)

### Theme 3 instrumental support

#### Support from the DFV sector

Women talked at length about the numerous bureaucratic hurdles involved in accessing housing support or refuges, especially in regional areas. Women impacted by economic abuse linked to their partner’s gambling noted the critical importance of having access to emergency funds to secure safe and stable accommodation. A few women related beneficial instances of emergency help and other interventions facilitated through local government-funded DFV services. One woman noted:

*“I had a lot of help from different emergency funds, for things like food and sometimes school fees and stuff when I have things sprung on me. I just didn’t have any savings.”* (Macy, WMG, age 30-39)

Women identified programs offering practical strategies and support as being particularly useful. Women were frequently coerced into handing over assets (for example, savings, jewellery, car and house) to fund their partner’s gambling habit, leaving them with no resources and trapped in violent situations. The following comments provide clear examples of how women benefited from practical support from DFV services, such as clothing and household goods, when they had decided to leave an abusive partner. This support was critical because *“if you’re fleeing domestic violence, you’re not necessarily going to leave with anything at all, except what’s on your back.”* (Shona, WWG, age 60–69). Furthermore, this woman also elaborated on how the service had helped her when in crisis mode:

*“... they (DFV service) paid for the train ticket for me to get to Brisbane. They paid for everything. It’s this type of support, the money, the practical stuff that’s most helpful when you are in crisis mode.”*

Safe refuge accommodation, material support for women in times of financial distress, supportive responses, and the availability of summary information collated from various relevant organisations were all identified as critical for women who had been left destitute by their partner’s gambling. Many women also commented on the usefulness of working through a safety plan and exit plan with service providers:

*“Yeah, the exit plan was most helpful. Even if it’s a one-sentence exit plan, there’s always some direction given, or provided, or to choose from, and it’s always prosperous…because obviously I’m moving forward, compared to where I would have been at the beginning.”* (Misty, WMG, age 30-39)

#### Support from the gambling help and financial counselling sector

Once they had discovered the extent of their partner’s gambling and economic abuse, many women worked on ways to protect their own money and the household’s finances. This was often extremely difficult or even impossible where their partner subjected them to violence if they did not give him full control over the family’s money. Gambling often results in economic abuse and hardship; therefore, numerous gambling help services employ specialist financial counsellors who are trained to identify and respond to economic abuse. With support from these services, some women opened new bank accounts, increased the security on their accounts, and limited their partner’s access to funds. These strategies helped several women to protect their finances when they were in the relationship.

Several women redirected their income into new accounts to keep it separate from their partner’s money. For example, one woman reported:

*“I separated my money. I opened up my own bank account. I’ve got my wages and my pension going into my own account in my own name but I still contribute to the bills…He was cranky and cross with me about that.”* (Rachel, WMG, age 50-59)

Other women strengthened the security of their accounts, with some working with banks and banking apps to do so. For example, one woman said: *“I’ve changed bank accounts with four different banks because of the security and I’ve told them what’s going on.”* (Helen, WMG, age 50–59). One young mother described strategies she had implemented while in a relationship with a man who had used her bank card for his poker machine gambling and online betting, stolen the Christmas money, and threatened her by choking and with a knife. Her priority was to ensure she could feed her infant son:

*“When he’s got paid it came in after midnight…so as soon as he walked out the door I would go online and transfer enough money for all the bills, you know, so he wouldn’t gamble all of it.”* (Harper, WMG, age 20-29)

For women impacted by violence linked to their own gambling problem, practical strategies offered by gambling help services (for example, support to identify triggers or minimise harm), were valued. One woman revealed how a gambling help service helped her navigate the self-exclusion process (where a person with a gambling problem voluntarily excludes themselves from specific gambling venues):

*“The venue is only 100m away, the local pub. So, I’m a member there…we identified this as a trigger, and she [gambling counsellor] was able to help me exclude myself, and from another pub, which worked for quite a while.”* (Stef, WWG, age 30-39)

In our study, economic abuse linked to gambling caused significant financial hardship. Working with gambling help and financial support services to identify and implement strategies to safeguard finances and manage debt was emphasised by many women.

## Discussion

This is the first in-depth qualitative study to explore the service experiences of women impacted by gambling related IPV. By focusing on positive experiences through a strengths-based approach, this study provides insight into what works for women. The findings highlight existing strengths and capacities and identify areas where further support is required. In short, the ways individual practitioners and services engage with women seeking help for the intersecting issues of gambling and IPV, and the protocols for screening, assessing and helping them are central to supporting and protecting women. Consistent with other research exploring IPV when other co-occurring health conditions are present [26, 31], this study found that women valued responses from practitioners and services that addressed their multiple, intersecting and complex needs. As well as needs arising from IPV victimisation, women impacted by gambling related IPV require support to cope with the emotional, relationship and psychological stress arising from their partner’s gambling. Men with gambling problems often have mental health and substance misuse problems, further exacerbating IPV and potentially leading to devastating consequences [5]. In addition, women with gambling problems also struggle with substance misuse problems, mental health and other comorbid issues [43]; it was not unusual for women in our study to find themselves in a relentless self-reinforcing cycle of gambling and abuse. Examining IPV through an intersectional lens [44], enables service providers to tailor support for victims with complex intersecting health issues.

Encouragingly, and in line with other studies [20, 26], routine inquiries and screening processes were valued by women, providing insight into the different ways that risk from problem gambling and violence manifest, and encouraging further disclosure. Whilst surveys show people are aware that a physical assault constitutes IPV [45], other behaviours such as economic abuse, or intimidating and controlling behaviours aimed at manipulating the woman into providing funds for gambling, may not be recognised as such. Our findings highlight the importance of screening for economic abuse, given its strong link with problem gambling and the unrecognised, underreported and invisible nature of this abuse [46–48]. Professional development for practitioners in IPV, gambling help and financial counselling could develop knowledge and skills to encourage screening for and responding to gambling related economic abuse. While our findings do support the use of structured screening tools, such tools must be short and practical to use, yet thorough enough to cover the risk [49]. Freytag and colleagues [8] recommend the ‘DOOR 1’ risk screen, a validated risk screening measure that takes clients 15 min to complete by tablet or paper, and covers around 100 risk items [50]. The DOOR 1 can be used as part of a collaborative interagency and case management approach by practitioners who are not DFV specialists.

Women in our study also appreciated timely referrals to other agencies within and outside the health sector. From a public health perspective, the importance of working beyond the health sector to improve health outcomes is well documented [51]. As Ragusa [52] highlights, understanding and improving service delivery for IPV demands an interdisciplinary approach considering the varied and complex factors involved. Front line practitioners, such as DFV counsellors, are vital in ensuring clients can disclose their abuse in a safe and supportive environment [31] and receive information regarding options available. However, financial concerns play a prominent role in women’s lives when struggling with the decision to leave or not, especially when gambling has depleted their financial resources [12, 14]. In recent years, the DFV field has increasingly recognised the importance of addressing women’s financial security [52, 53]; this is especially relevant when working with women impacted by violence related to gambling. Gambling help and financial counsellors are uniquely positioned to identify and deal with the fallout from gambling related IPV [8]; in Australia, economic abuse casework is typically an important part of the role [54]. In our study, referrals to gambling help and financial support services helped women alleviate monetary challenges, gain insights into their situation, manage debt, and safeguard finances. These findings reinforce the importance of well-linked service provider networks that transcend traditional health sector responses.

Problem gambling and IPV are both highly stigmatised and associated with negative stereotypes [14, 55, 56], hence practitioners need to be aware of the double stigma facing women impacted by gambling related IPV. Women with a gambling problem face intense stigma; high levels of damaging self-stigma are evident in this group [57]. In our interviews, multiple aspects of stigma were uncovered, leading to women feeling ashamed and inadequate, compounding emotional and social isolation. Our study supports evidence from other research that women impacted by highly stigmatised health issues value a non-judgmental, trauma-informed approach, tailored to their needs [19, 31]. Support for women needs to be non-judgmental due to the stigmatising nature of both problem gambling and IPV [12], and trauma-informed due to their varying and intersecting needs [20]. Sharing lived experiences through group counselling or support groups, was a crucial part of the healing process for many women and has been shown to an effective in other settings [58, 59], especially for highly stigmatised issues. Cultural, anticipated and internalised stigma can deter help-seeking [24, 25]; hence practitioners working with women must address the many facets of the woman’s complex situation and the shame associated with the struggle to move forward.

As noted by Freytag and colleagues [8], therapeutic approaches should never oversimplify a woman’s predicament or perpetuate misguided information such as “Why doesn’t she just leave him?” or “She should just stop gambling!”. Importantly, practitioners also need to consider whether strategies promoted to assist women dealing with gambling related IPV may increase the risk of harm. For example, when working with women who gamble, it is crucial to understand the role gambling plays. For some women, gambling venues are seen as accessible, safe and welcoming spaces, and may provide respite from their partner’s violence [60]. Electronic gaming machines (EGMs), in particular, are highly accessible and attractive to women as they facilitate dissociation, and help women extend time away from pain, worries and difficult realities [61, 62]. Furthermore, lack of financial independence traps women in violent relationships [63, 64]; gambling provides one of the few sources of hope for women in these situations. Supporting women impacted by gambling related IPV requires a multi-pronged approach that addresses the harms caused by IPV and problem gambling. Counselling, therefore, needs to focus on expanding a women’s coping capacity and exploring safer respite options.

It is important to note that women escaping gambling related IPV have safety needs and complex financial needs (resulting from gambling related economic abuse or because funds have been depleted through their own gambling). Consistent with the broader DFV literature [10], women highlighted the importance of having access to safe shelters, more affordable and stable housing, and emergency funds. Accessing affordable, permanent housing is critical to the long-term safety and well-being of women and their families [65]. Encouragingly, access to safe places and emergency accommodation for women impacted by IPV is prioritised in the Australian “National Plan to Reduce Violence against Women and their Children 2010–2022” [66], a key document outlining government regulations and responses to DFV. Also consistent with findings from other research [67, 68], women highlighted the importance of developing a tailored safety plan focused on the woman’s unique life circumstances and plans. Safety planning aims to collaborate with women to help them identify acceptable and feasible options to increase their safety and decrease their exposure to harm [69]. Strategies that don’t match risks and circumstances may not improve safety and may increase risk [68].

Problem gambling has devastating financial consequences and creates additional financial stressors for women and their families [14, 70], hence instrumental support from financial counsellors and gambling help was seen as critically important. Evidence suggests that practical support, aimed at encouraging women to manage their finances, improve economic empowerment and build self-efficacy in a way that minimises the risk of exploitation, can be used as a mechanism to address and prevent further abuse [53, 71]. Women were positive about the assistance received from financial counsellors; strategies such as securing loans to pay off debts and practical strategies to safeguard finances such as opening new bank accounts or strengthening the security of their accounts helped protect the household’s money. For women seeking help for IPV related to their own gambling, practical strategies and support from gambling help were valuable. In addition, services can assist women to use concrete tools such as voluntary self-exclusion, designed to limit access to gambling. By increasing the likelihood of reductions in gambling behaviour, problem gambling symptomatology, gambling urges and gambling harm, self-exclusion can be an important adjunct to treatment [72–74].

### Limitations

This study provides a unique insight into the service experiences of Australian women impacted by gambling related IPV, however, the study is not without its limitations. Women were purposefully sampled to explore and unpack their experiences of IPV victimisation linked to gambling; the researchers recognise that this study does not represent the service experiences of all women impacted by gambling related IPV. Although women who participated were from diverse backgrounds, the relatively small sample size did not allow analysis by characteristics such as race or ethnicity. Future research would benefit from exploring the service experience of women from a different cultural perspective. Exploring experiences through a cultural lens will help support the design and delivery of culturally and linguistically appropriate support services for women impacted by gambling related IPV. Though women are the focus of this paper, the service needs of children in this family situation cannot be overlooked. Further research could integrate the voices of children impacted by gambling related IPV and identify effective service responses for women and their children.

## Conclusion

IPV and problem gambling are both common and harmful public health problems. Effective service responses depend on understanding how problem gambling and IPV intersect. Supporting women impacted by gambling related IPV requires a multi-faceted, multi-agency approach that reduces both problem gambling and IPV. Violence against women inextricably tied to problem gambling is a dangerous combination, yet, as this study demonstrates, positive responses from health and social services provide a valuable way forward for improving women’s lives.

## Data Availability

The study datasets contain sensitive personal information and are held on a secure cloud-based server with restricted access. Access requires the approval of the ethics committee and data custodians. The corresponding author can be contacted for data requests.

## Abbreviations

AIHW: Australian Institute Health & Welfare
DFV: Domestic & Family Violence
EGM: Electronic Gaming Machine
IPV: Intimate Partner Violence
NDV: Non-Domestic Violence
WHO: World Health Organisation
WMG: Woman impacted by male partner’s gambling
WWG: Woman impacted by woman’s gambling
